# Systemic Sclerosis and Systemic Lupus Erythematosus Overlap Syndrome with Pulmonary Arterial Hypertension Successfully Treated with Immunosuppressive Therapy and Riociguat

**DOI:** 10.7759/cureus.4327

**Published:** 2019-03-26

**Authors:** Kentaro Kuzuya, Soichiro Tsuji, Masato Matsushita, Shiro Ohshima, Yukihiko Saeki

**Affiliations:** 1 Rheumatology and Allergology, National Hospital Organization Osaka Minami Medical Center, Osaka, JPN

**Keywords:** mixed connective tissue disease (mctd), systemic sclerosis (ssc), pulmonary arterial hypertension (pah), riociguat, overlap syndrome (overlap)

## Abstract

We report the case of a 40-year-old patient with systemic sclerosis (SSc) and systemic lupus erythematosus (SLE) overlap syndrome with pulmonary arterial hypertension (overlap-PAH) that was successfully treated with a combination of immunosuppressive therapy and the soluble guanylate cyclase stimulator riociguat. She was diagnosed with mixed connective tissue disease (MCTD) two years prior to admission. She was admitted to our hospital with dyspnea on exertion and progressive skin sclerosis. She fulfilled both SLE and SSc classification criteria and was re-diagnosed with overlap syndrome. The tricuspid valve pressure gradient (TRPG) on echocardiography was 64 mmHg at admission. On right heart catheterization, mean pulmonary arterial pressure (mPAP) was 43 mmHg and pulmonary capillary wedge pressure was 15 mmHg. We diagnosed her with SSc-SLE overlap-PAH and started treatment with corticosteroids and intravenous cyclophosphamide. We also started treatment with riociguat because we speculated she had a component of SSc-PAH and that immunosuppressive therapy alone may be insufficient. We chose riociguat because of its favorable treatment effect on SSc-PAH. Two months after treatment, her TRPG improved to 33 mmHg and the skin sclerosis improved dramatically, suggesting the efficacy of multi-drug treatment and the importance of early intervention.

## Introduction

Connective tissue disease-associated pulmonary arterial hypertension (CTD-PAH), categorized as group I pulmonary hypertension (PH) [1], is often life-threatening. CTD-PAH sometimes responds to immunosuppressive therapy [2-4]. However, patients with CTD-PAH have a lower survival rate than those with whole group I PH [5]. A previous study demonstrated that patients with systemic sclerosis-associated pulmonary arterial hypertension (SSc-PAH) have a lower survival rate, while those with systemic lupus erythematosus-associated PH (SLE-PAH) have a similar survival rate as those with idiopathic PAH [6]. This is possibly because SSc-PAH is unresponsive to immunosuppressive therapy, is less responsive to PAH-specific therapy, and has other factors that affect prognosis, such as the component of interstitial lung disease and/or left heart disease-associated PH (LHD-PH). The prognosis of mixed connective tissue disease-associated PAH (MCTD-PAH) is not seen in large cohorts because of the disease’s rarity; however, a MCTD-PAH group had significantly worse five-year survival than a MCTD-non-PAH group [7] and PAH has been the main cause of death in patients with MCTD to date. Cases of overlap syndrome-associated PAH (overlap-PAH) have rarely been reported. Here we report the case of an SSc-SLE overlap syndrome patient with severe PAH that was successfully treated with immunosuppressive therapy and riociguat, a soluble guanylate cyclase stimulator.

## Case presentation

The patient was a 40-year-old woman who had been diagnosed with MCTD because of Raynaud’s phenomenon, swollen fingers, heartburn, polyarthralgia, and a high anti-U1RNP antibody titer two years before admission. Echocardiography performed in the same year of diagnosis was normal, so she had been taking no medication except nonsteroidal anti-inflammatory drugs for polyarthralgia.

She developed dyspnea on exertion and skin sclerosis after she became pregnant, one year and three months before admission. She thought that these symptoms were caused by the pregnancy. She delivered via cesarean section five months before admission, but her symptoms worsened later. She also began experiencing bilateral leg muscle pain one month before admission. On laboratory examination, thrombocytopenia (11.4 × 104/μL), hypocomplementemia (C3, 68 mg/dL), elevated CPK (981 IU/L), elevated NT-proBNP (614 ng/dL), and proteinuria (urinary total protein to urinary creatinine was 1.04 g/g creatinine) were detected one week before admission. On immunological examination, anti-U1RNP antibody, anti-DNA antibody, and anti-Sm antibody were positive, while anti-PM/Scl-100 antibody and anti-Th/To antibody were slightly positive (immunoblot assay). On pulmonary function test, vital capacity as percent of predicted (%VC) was 71%, forced expiratory volume in 1 s as percent of predicted (FEV1.0%) was 86%, and diffusing capacity of carbon monoxide/vital capacity as percent of predicted (%DLCO/VA) was 69%. On echocardiography, the left ventricular ejection fraction was 69% and tricuspid valve pressure gradient (TRPG) was 64 mmHg with no other ventricular regurgitation or stenosis; dilatation of the right atrium and ventricle and exclusion of the left ventricle at the early diastolic phase were detected. On the left ventricular (LV) inflow velocity pattern, deceleration time was 160 ms and E/A was 0.85. On the tissue Doppler imaging (TDI), e′ at the septal/lateral side were 6.9/14.4 cm/s. E/e′ at the septal/lateral side was 7.9/3.8 cm/s. 

Connective tissue disease-associated PAH was suspected, for which she was admitted to our hospital. Her height and weight were 155 cm and 43 kg, respectively. Her vital signs were normal. A physical examination revealed ankyloglossia, bilateral leg muscle tenderness, and skin sclerosis that had spread to her face, neck, and both hands from the fingers to the forearms; the modified Rodnan’s skin score (mRSS) was 21 points [8]. The manual muscle testing (MMT) results were as follows: deltoid, 4/4; biceps, 3/4; iliopsoas, 5/5; quadriceps, 5/5; hamstring, 4/4; anterior tibia, 5/5; and gastrocnemius, 4/4. Dermoscopy revealed microvascular hemorrhage in the nailfold capillaries. The six-minute walk test (6MWT) was 240 m. The chest thoracic ratio (CTR) on the chest radiograph was 44% compared with 36% two years before admission. Chest computed tomography detected no interstitial lung disease or pulmonary thromboembolism. The ventilation and perfusion scintigraphy were normal. The short-tau inversion recovery (STIR) sequence of MRI of the bilateral leg muscles showed high signal intensity. The results of right heart catheterization were: mean pulmonary arterial pressure (PAP), 43 mmHg (systolic/diastolic pressure: 70/28 mmHg); pulmonary artery wedge pressure (PAWP), 15 mmHg; diastolic pressure gradient (DPG), 13 mmHg; pulmonary vascular resistance index, 839 dynes/s/cm-5/m2 (normal range: 200-400); and cardiac index (CI), 2.67 L/min/m2. 

**Figure 1:**
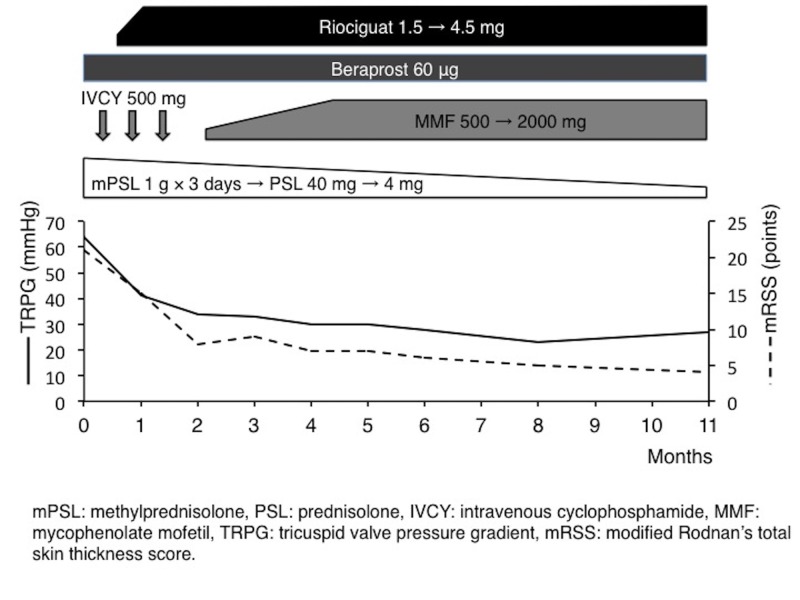
The patient’s one-year clinical course.

The patient fulfilled the SLE classification criteria [9], and we started treatment with corticosteroids and intravenous cyclophosphamide. We also started riociguat two weeks after admission because she also fulfilled SSc classification criteria [10] and we speculated that immunosuppressive therapy alone is insufficient because her PAH was derived from components of both SLE and SSc. After two months of treatment, her symptoms and laboratory data improved: CPK declined to 31 IU/L, C3 increased to 104 mg/dL, NT-pro BNP declined to 109 ng/dL, urinary total protein to urinary creatinine declined to 0.43 g/g creatinine, CTR on the chest radiograph declined to 37%, TRPG declined to 33 mmHg on follow-up echocardiogram, 6MWT improved to 400 m, and WHO-FC improved to class I; after this time, she was discharged. Although she could continue taking maximum-dose riociguat, she could not continue the cyclophosphamide pulse therapy after the third course due to acute reactions (fever and arthralgia). Therefore, we started mycophenolate mofetil. Her TRPG declined to 28 mmHg six months after treatment and her condition remained stable for one year. Her skin sclerosis continued improving dramatically (Table 1, Figure 1).

**Table 1:** Laboratory data on admission. WBC, white blood cell; Neu, neutrophil; Ly, lymphocyte; RBC, red blood cell; Hb, hemoglobin; MCV, mean corpuscular volume; PLT, platelet; LAC, lupus anticoagulant; Alb, albumin; T-Bil, total bilirubin; LDH, lactose dehydrogenase; AST, aspartate transaminase; ALT, alanine transaminase; BUN, blood urea nitrogen; Cre, creatinine; UA, uric acid; Na, serum sodium; K, serum potassium; Cl, serum chloride; APTT, activated partial thromboplastin time; PT-INR, international normalized ratio of prothrombin time; CRP, C-reactive protein; NT-proBNP, N-terminal pro-brain natriuretic peptide; TSH, thyroid stimulating hormone; FT4, free thyroxine; C, complement; CH50, hemolytic complement activity; C1q, immune complex; Ig, immunoglobulin; ANA, anti nuclear antibody; H. pylori, helicobacter pylori; DRVVT, Russell's viper venom time; KCT, kaolin clotting time; U1RNP, U1 ribonucleoprotein; Ab, antibody; ds-DNA, double-stranded DNA; Sm, Smith; Scl, scleroderma; RNApIII, RNA polymerase 3; MDA-5, melanoma differentiation associated gene 5; ARS, aminoacyl transfer RNA synthetase; PA-IgG, platelet associated antibody IgG; UTP/UCre, urine protein to creatinine ratio.

Blood tests					
WBC	5430 /μL	APTT	36.1 s	Anti-Sm Ab	39.4 IU/mL
Neu	2850 /μL	PT-INR	1.00	Anti-Scl70 Ab	<1.0 IU/mL
Ly	1970 /μL	Fib	192 mg/dL	Anti-centromere Ab	<2.0 IU/mL
RBC	5.47 × 10^6^ /μL	D-dimer	<0.5 μg/mL	Anti-RNA-pIII Ab	<5 IU/mL
Hb	12.9 g/dL	LAC (DRVVT)	1.06	Anti-Mi2 Ab	<5 IU/mL
MCV	76.1 fL	LAC (KCT)	1.10	Anti-TIF1γAb	<5 IU/mL
Plt	11.4 × 10^4^ /μL	Direct Coombs	Negative	Anti-MDA5 Ab	<5 IU/mL
Alb	3.5 g/dL	Haptoglobin	97 mg/dL	Anti-ARS Ab	<5 IU/mL
T-Bil	0.69 mg/dL	CRP	0.04 mg/dL	Anti-PM/Scl100 Ab	±
LDH	354 IU/L	NT-proBNP	614 ng/dL	Anti-Th/To Ab	±
AST	62 IU/L	C3	68 mg/dL	Anti-cardiolipin Ab	<8.0 IU/mL
ALT	26 IU/L	C4	13 mg/dL	Anti-β2GPI Ab	<0.7 IU/mL
CPK	981 IU/L	CH50	40.0 mg/dL	H. pylori IgG	<10 IU/mL
BUN	8.4 mg/dL	C1q	<1.5 μg/mL	PA-IgG	<14.7 ng/10^7^ cells
Cre	0.39 mg/dL	IgG	3046 mg/dL	Urinary sediment	
UA	3.3 mg/dL	IgA	300 mg/dL	RBC	1-4 /HPF
Na	138 mEq/L	IgM	170 mg/dL	WBC	1-4/ HPF
K	3.7 mEq/L	ANA	×2560 Speckled	Casts	Negative
Cl	103 mEq/L	Anti-U1RNP Ab	1250 IU/mL	UTP/UCre	1.04 g/gCre
Glucose	87 mg/dL	Anti-DNA Ab	9 IU/mL		

## Discussion

Alharbi et al. reported that the SSc-SLE overlap syndrome group was significantly younger at diagnosis and more frequently had PAH than the pure SSc group [11]. Thus, we should carefully monitor for PAH in SSc patients with overlap syndrome. The diagnostic conversion of MCTD into the other CTD such as SLE or SSc is not rare in the long-term follow-up [12]. This patient showed disease conversion from MCTD to SSc-SLE overlap syndrome two years after the first diagnosis. It was recently reported that the anti-U1RNP-positive group of SSc-PAH had a better survival rate than the negative group [13]. In this study, patients with SSc-SLE overlap syndrome were included in the SSc group, so a good response to immunosuppressive therapy may have contributed to better survival of the anti-U1RNP-positive group of SSc, such as this case. Her proteinuria may have been due to lupus nephritis because it was gradually normalized with complement and platelet count after starting immunosuppressive therapy, although we did not perform a renal biopsy. She also had a component of polymyositis because she had no rash, decreased MMT, elevated CPK, high signal intensity in the bilateral leg muscles on the STIR sequence of MRI, and a good response to immunosuppressive therapy. Using classification criteria [14], she was classified as having possible polymyositis, but we did not perform a muscle biopsy. However, idiopathic inflammatory myositis-associated PAH without extensive interstitial lung disease is extremely rare. 

It is unknown whether MCTD or overlap-PAH consists of mixed pathology of SLE and SSc-PAH or its specific pathology. Sasaki et al. reported that histopathological findings in autopsy cases of MCTD in which the cause of death was PAH were severe fibrous intimal thickening, occlusion, and thrombosis of the pulmonary arteries. On the other hand, fibrinoid vasculitis was rarely seen [15]. These findings may indicate that severe MCTD-PAH has at least some factors of vasculopathy that resemble SSc-PAH, although the treatment effect on pathological findings was not considered because immunosuppressive therapy before death might diminish the findings of vasculitis.

Because the PAWP at admission (15 mmHg) was on the borderline to divide patients into group I and group II pulmonary hypertension [16], we should judge whether she has a component of LHD-PH arose from the myocardial fibrosis of SSc. The results of the echocardiogram at admission revealed that she had a preserved EF, normal E/e′, no ventricular disease, and no congenital heart disease. Although her E/A ratio was 0.85 and improved to 1.27 after one year, this rather means improvement of reduced LV filling due to the pre-capillary PH than LV diastolic dysfunction. Thus, we speculate that post-capillary PH due to LHD was not clinically evident in this patient.

We chose riociguat because it had the following favorable factors for SSc: the SSc-PAH subgroup in a phase 3 trial of riociguat showed a three-year survival rate equal to that in the entire group I PH [17], riociguat reduced skin thickening in a SSc mouse model, and its effect was significantly stronger than that of sildenafil [18] and riociguat, but sildenafil did not reduce right ventricular fibrosis in a PAH mouse model [19]. The patient’s skin sclerosis continued improving dramatically after continuing riociguat for one year (Figure 1), suggesting a possible anti-fibrotic effect. However, we cannot declare that this effect is due to riociguat because we also used other immunosuppressive therapies such as corticosteroids and cyclophosphamide. We also did not conduct a skin biopsy so pathological changes were not evident and no other clear symptoms of SSc were observed (heartburn had already improved after proton pomp inhibitor administration at admission). Thus, the accumulation of cases is needed to elucidate the anti-fibrotic effect of riociguat. A phase II trial of riociguat for diffuse cutaneous SSc (RISE-SSc), with its primary endpoint being mRSS, is now ongoing and a favorable outcome is expected. 

The only immunosuppressive treatments that are effective for CTD-PAH as induction therapy are cyclophosphamide and corticosteroids [2-4]. There is no consensus about whether we should start immunosuppressants in the maintenance phase. We recommend immunosuppressant in addition to corticosteroid because CTD-PAH followed by corticosteroid monotherapy tends to relapse and require retreatment according to a past report [3]. Another possible factor that may have contributed to a favorable outcome in this patient is that the intervention was delivered relatively early; past reports showed that WHO-FCIII or IV was a risk factor for nonresponse [2-3]. 

Although we are under the impression that SLE or MCTD-PAH is responsive to immunosuppressive therapy, Sanchez et al. reported that immunosuppressive therapy alone is effective in only about 40% cases of SLE or MCTD-PAH, and no cases of SSc-PAH had responded [2]. These are the reasons why we should consider the possibility that immunosuppressive therapy alone is not enough in some cases and start treating with PAH-specific therapy as soon as possible to intervene before the vascular injury becomes irreversible. 

## Conclusions

Combination therapy with immunosuppressants and vasodilators is recommended in cases of SSc-SLE overlap-PAH. Riociguat is suggested to treat SSc-PAH or overlap-PAH with a component of SSc, such as this case, because of the favorable effects of treatment on both PAH and skin sclerosis.
